# Exploring Motivations and Barriers To Accreditation Adoption Among Physician Assistant and Nurse Practitioner Emergency Medicine and Orthopedic Surgery Residency and Fellowship Programs

**DOI:** 10.7759/cureus.36490

**Published:** 2023-03-21

**Authors:** Vasco Deon Kidd

**Affiliations:** 1 Orthopedic Surgery, School of Medicine, University of California, Irvine, USA

**Keywords:** accreditation and assessments, postgraduate np pa training, transition-to-practice, nurse practitioner, residency program, fellowship training, advanced practice provider, physician associate, physician assistant, quality and accreditation

## Abstract

Although there has been a rapid increase in the number of physician assistant (PA) and nurse practitioner postgraduate residency/fellowship specialty training programs in the United States, voluntary accreditation of these programs is a relatively new phenomenon. There is little research examining which factors may be predictors of accreditation adoption among PA and nurse practitioner (NP) postgraduate programs. Therefore, the primary aim of this study was to investigate which motivating factors influence postgraduate programs choice on whether to pursue accreditation.

Methodology

A descriptive cross-sectional survey consisting of 10 questions was distributed to 56 postgraduate emergency medicine and orthopedic surgery PA, NP, and joint PA/NP residency/fellowship programs via email between November 2022 and February 2023. Descriptive statistics were performed.

Results

Nineteen postgraduate programs responded to the survey. Two programs submitted incomplete surveys where less than 50% of the total items were completed. The partially completed surveys were omitted from the data analysis. The final response rate was 30% (17/56). Among the responded programs, 47% (8/17) offer dual PA and NP training, and 53% (9/17) are PA only. Although 88% (15/17) of postgraduate programs have discussed accreditation funding with their sponsoring institution only 76% are planning to do so or are in the process of pursuing accreditation.

The two most common reasons for pursuing accreditation are program validation and assessment of educational quality 59% (10/17) and competition for applicants 24% (4/17). However, Magnet hospital designation, requirement of the sponsoring institution, or demand from employers were not determining factors of whether to pursue accreditation or not. Reasons for not pursuing accreditation included high costs of accreditation 18% (3/17), lack of perceived value of accreditation 6% (1/17), and lack of protected time to pursue accreditation 6% (1/17).

Conclusion

Although literature is still lacking on the effectiveness and impact of PA and NP postgraduate program accreditation, the findings of this rare study indicate that postgraduate programs are motivated to pursue accreditation. It is important for accreditors to communicate the utility of program-level accreditation and other value-added benefits to programs and their sponsoring institutions. Further work is necessary to better understand the value of external validation, specific drivers for validation, and barriers that influence accreditation adoption among these postgraduate programs.

## Introduction

Physician assistant (PA) postgraduate training began in the late 20th century while the first nurse practitioner (NP) residency started in 2007. Over the last decade, there has been a dramatic expansion of postgraduate PA and NP training programs in the United States [1-3]. To date, there is a lack of consensus regarding the designation used to refer to these programs with the term residency and fellowship used interchangeably. Additionally, the number of PA and NP entry-level graduates each year far exceeds the current supply of PA and NP postgraduate residency/fellowship positions. Postgraduate programs offer formalized training designed to increase specialty-specific knowledge, skills, competence, and facilitate role transition. This is consistent with a prior study, which found that nurse practitioners who attended a primary care residency program reported enhanced practice autonomy, interprofessional collaboration, job satisfaction, and lower job turnover than those without residency training [4]. There is no equivalent study comparing PA postgraduate specialty training with PAs who did not participate in residency training.

Postgraduate program length is typically 12 months, with some programs spanning 24 months. While some NP residency/fellowship programs receive federal funding, most PA and NP postgraduate programs are self-funded. Postgraduate programs may choose to voluntarily apply for accreditation from one of several accrediting bodies, the Accreditation Review Commission on Education for the Physician Assistant (ARC-PA), the Consortium for Advanced Practice Providers (CAPP) formally known as National Nurse Practitioner Residency and Fellowship Training Consortium (NNPRFTC), the American Nurses Credentialing Center Advanced Practice Provider Fellowship Accreditation (ANCC APPFA), and the Commission on Collegiate Nursing Education (CCNE). Among accreditors, there is variation in accreditation requirements and standards making it very difficult to compare outcome measures across postgraduate programs accredited by different agencies. Additionally, CAPP and APPFA offer accreditation for postgraduate NP, PA, and joint PA/NP training programs [5,6]. Moreover, the Accreditation Commission for Education in Nursing (ACEN) and ARC-PA in a new partnership announcement will offer joint PA/NP accreditation [7]. Joint accreditation is important since approximately half of postgraduate programs accept both PAs and NPs [8]. Some benefits of joint accreditation include decreased administrative burdens and costs for postgraduate programs during the initial application and renewal process, having one set of standards for an all-inclusive specialty training experience, and meeting the unique needs of the healthcare institutions, such as enhancing multifaceted, diverse learning experiences [9,10].

Although there has been an uptick in the number of accredited PA and NP postgraduate programs, concerns persist regarding the value of accreditation. There is insufficient evidence to assess the organizational value and impact of PA and NP residency/fellowship program accreditation [11]. Moreover, there are no published large-scale empirical research studies measuring accredited PA and NP postgraduate program outcomes against clinical outcomes.

Furthermore, others may argue whether accreditors have sufficient content expertise and resources to evaluate specialty-specific competency-based curricula delivered to licensed PAs and NPs enrolled in postgraduate specialty training. Despite these considerations, it has been surmised that accreditation will enhance program standardization and improve program quality and curricular effectiveness [12,13]. This is especially important given the significant variability across postgraduate programs in terms of program length, curricular elements, administrative structure, procedural competencies, instructional characteristics, and evaluation methods underscoring the challenge in comparing outcomes of PAs and NPs enrolled in postgraduate training [1,3,14]. Therefore, the primary aim of this study was to investigate, which motivating factors influence postgraduate programs' choice on whether to pursue accreditation.

## Materials and methods

University of California, Irvine, issued approval with IRB #2325 self-exempt. A comprehensive list of PA, NP, and joint PA/NP postgraduate emergency medicine and orthopedic surgery residency/fellowship programs was compiled from the following sources: the Association of Postgraduate PA Programs, the Association of Postgraduate Advanced Practice Registered Nurse (APRN) Programs (APGAP), and programs listed on the American Academy of Emergency Nurse Practitioner’s (AAENP) website and from a recently published article on PA and NP postgraduate orthopedic surgery programs [15]. These two specialties were included because they represent the largest number of postgraduate programs in the United States. A web-based cross-sectional descriptive survey was sent to 58 postgraduate programs. Two emails bounced back or failed to deliver, resulting in a total of 56 valid emails. The email invitation to the electronic survey explained the purpose of the study and assured participants about confidentiality. The study period was from November 2022 to February 2023. The survey consisted of 10 items (nine closed-ended and one open-ended questions). This study used pre-existing and pre-tested survey questions from the author’s previously published articles. Some of the questions were slightly modified to help address the research question. Six email reminders were sent to non-respondents over the study period to encourage survey participation. The average length of time to complete the survey was less than 3 min. Survey data was aggregated, and descriptive statistical analyses were conducted using the statistical package embedded within the Qualtrics survey software (Provo, UT: Qualtrics). When calculating sample size, it is estimated that 49 (87.5%) or more survey responses are needed to have a confidence level of 95% within a 5% margin of error.

## Results

Nineteen postgraduate programs responded to the survey. Two programs submitted incomplete surveys where less than 50% of total items were completed. The partially completed surveys were omitted from data analysis. The final response rate was 30% (17/56). Among the responded programs, 47% (8/17) offer dual PA and NP training, and 53% (9/17) are PA only. Eleven emergency medicine (65%) and six orthopedic programs (35%) responded to the survey. Postgraduate programs located at academic medical centers were 47% (8/17), multi-hospital systems were 29% (5/17), county hospitals were 12% (2/17), and others (unidentified) were 12% (2/17). Of those who completed the survey, 76% (13/17) were PAs, 18% (3/17) NPs, and 6% (1/17) were physicians.

Overwhelmingly, program directors who responded to the survey were 94% (16/17), whereas 6% (1/17) were others. Although 88% (15/17) of postgraduate programs have discussed accreditation funding with their sponsoring institution only, 76% (13/17) are planning or in the process of pursuing accreditation. Of those postgraduate programs pursuing accreditation, seven PA-only postgraduate programs will apply to the ARC-PA, and five joint PA/NP postgraduate programs will apply to ANCC APPFA, and one joint program will apply to both ANCC APPFA and ARC-PA. What is not clear from the data is what motivates a postgraduate program to select a specific accrediting agency. The variables that influence a postgraduate program's decision to pursue accreditation included program validation and assessment of educational quality 59% (10/17), and competition for applicants 24% (4/17) (Table 1).

**Table 1:** Reasons for pursuing accreditation among postgraduate programs.

Reasons for pursuing accreditation	Number of responses, n% = 17
Program validation and assessment of educational quality	59% (10/17)
Competition for applicants	24% (4/17)
Other (indeterminate response)	18% (3/17)

However, Magnet hospital designation, requirement of the sponsoring institution, and demand from employers were not determining factors of whether to pursue accreditation. Reasons for not pursuing accreditation included, high costs of accreditation 18% (3/17), lack of perceived value of accreditation 6% (1/17), and lack of protected time to pursue accreditation 6% (1/17) (Table 2). The selected comments from respondents regarding accreditation are presented in Table 3.

**Table 2:** Reasons for not pursuing accreditation among postgraduate programs.

Reasons for not pursuing accreditation	Number of responses, n% = 17
High costs of accreditation	18% (3/17)
Lack of perceived value of accreditation	6% (1/17)
Lack of protected time to pursue accreditation	6% (1/17)

**Table 3:** Respondent quotes regarding accreditation.

Selected respondent quotes
“We are considering accreditation; however, it is cost prohibitive. There should be one set of national standards and some institutional financial benefit since they are not tuition-bearing programs.”
“Assuring standardization of quality among all postgraduate programs, similar to physician training programs.”
“At some point, risk management at the hospital will find out that the residency program could be accredited, and then they will demand that we follow (through) with it.”

Although not part of the present study, Table 4 provides a detailed snapshot illustrating the current number of accredited postgraduate residency/fellowship programs including applicant programs by accreditor. There appears to be demand for accreditation among postgraduate programs, which may increase competition among accreditors for market share. Having accreditation options allows postgraduate programs to select an accreditor that is best suited to meet their specific needs. The following accrediting agencies have received recognition (Table 5).

**Table 4:** Voluntary accreditation options for postgraduate physician assistant and nurse practitioner residency/fellowship programs. ANCC: American Nurses Credentialing Center; APPFA: Advanced Practice Provider Fellowship Accreditation; PTAP: Practice Transition Accreditation Program; CAPP: Consortium For Advanced Practice Providers; ARC-PA: Accreditation Review Commission on Education for the Physician Assistant; CCNE: Commission on Collegiate Nursing Education; NP: nurse practitioner; PA: physician assistant *ACEN/ARC-PA: the Accreditation Commission for Education in Nursing/the Accreditation Review Commission on Education for the Physician Assistant

Accrediting agencies	Accredits postgraduate PA programs	Accredits postgraduate NP programs	Accredits postgraduate joint PA/NP programs	Number of current accredited postgraduate programs, including applicant programs as of February 3, 2023, if available
ANCC APPFA	Yes	Yes	Yes	8 new applicant programs under APPFA. There are 26 NP postgraduate programs accredited under the ANCC PTAP program. These 26 programs will transition to APPFA over the next four years
CAPP	Yes	Yes	Yes	24 programs have been accredited by the Consortium, 6 programs awaiting accreditation decisions, and 8 programs waiting to schedule their site visits
ARC-PA	Yes	No	Expected sometime in 2023*	12 accredited PA fellowship/residency programs
CCNE	No	Yes	No	12 organizations in applicant status for accreditation of their nurse practitioner fellowship/residency programs

**Table 5:** Recognition of accrediting agencies. ANCC: American Nurses Credentialing Center; CAPP: Consortium for Advanced Practice Providers; ARC-PA: Accreditation Review Commission on Education for the Physician Assistant; CCNE: Commission on Collegiate Nursing Education

Accrediting agency	Recognition
ANCC	Recognized by ISO (International Organization for Standardization), 9001:2015 certification
CAPP	Recognition by the U.S. Department of Education
ARC-PA	Recognition by the Council for Higher Education Accreditation
CCNE	Recognition by the U.S. Department of Education

## Discussion

To my knowledge, this is the first attempt to evaluate which factors may be predictors of accreditation adoption among emergency medicine and orthopedic surgery postgraduate PA and joint PA/NP programs. The findings of this study, albeit on a small scale, seem to suggest that there is an interest among some postgraduate programs to improve their programs and educational quality. Competition for applicants is an additional factor that programs consider as they seek accreditation. Moreover, the findings indicate that Magnet hospital designation, requirement of the sponsoring institution, or demand from employers were not factors impacting a postgraduate program’s decision to pursue accreditation. Historically, graduation from unaccredited postgraduate programs has not affected income or employment rates for trainees [16]. Furthermore, the present study found that Magnet recognition was not a primary reason for pursuing accreditation. This finding corroborates previous research, which found that Magnet recognition had no impact on whether a transition-to-practice program (TTP) pursued accreditation [11]. It is also evident from the current study that exorbitant accreditation fees have become, first and foremost, a barrier to entry for some postgraduate programs. Fees vary across accreditors and multi-track postgraduate programs pay substantially more to achieve accreditation than single-track programs. Recognition of cost burden associated with PA and NP postgraduate training is critical since there is significant variability in individual program expenses and revenues. Anecdotally, many postgraduate programs struggle to generate enough clinical revenue to offset training at much less accreditation costs. Achieving accreditation can also increase demands on staff time and other resources, though the long-term benefits of accreditation may far outweigh the initial investment [17].

Nevertheless, there may be several reasons why postgraduate programs are unable to pursue accreditation. For those programs, the Society of Emergency Medicine Physician Assistants (SEMPA) jointly with postgraduate program directors have developed emergency medicine postgraduate training program standards, which were updated in August 2021 [18,19]. These comprehensive standards provide guidance on all aspects of emergency medicine postgraduate training. Also, as part of its membership criteria, APPAP provides guidance regarding program development and optimizing postgraduate education, curricula, operational policies, and assessment activities [20]. That said, given the rapid expansion in the number of PA and NP residency/fellowship training slots and the development of new postgraduate programs in the pipeline, accreditation is likely to become commonplace in the not-too-distant future.

Potential areas for future research

This study sheds some light on accreditation adoption among PA and joint PA/NP postgraduate programs. But, future research must be done to understand faculty and trainee attitudes and perceptions of the impact of accreditation, perceived readiness to pursue accreditation, costs and benefits associated with initial and re-accreditation activities, including maintenance fees in order to determine the return on investment [21]. Lastly, research is needed to explore which factors influence a postgraduate program’s choice in selecting a specific accrediting agency.

Limitations

This study is not without limitations. First, this study focused on emergency medicine and orthopedic surgery postgraduate PA and joint PA/NP residency/fellowship programs. Therefore, the findings are not generalizable to all postgraduate training programs. Second, this study includes a low response rate of 30% (17/56), which may have contributed to response bias. Low response rates are common with web-based surveys [22]. Consequently, postgraduate program surveys have historically low and variable response rates. Third, overrepresentation of emergency medicine programs may also have led to some degree of bias. Fourth, NP fellowship programs did not participate in the study, and their perspectives could have potentially influenced the results. Fifth, this study did not examine postgraduate programs that were early adopters of accreditation. This is a potential area of future research using a mixed-method study design. Sixth, the modified survey instrument used, although informed by prior research, did not undergo field testing. Therefore, study findings need to be interpreted with caution due to study limitations.

## Conclusions

Although questions about the value, effectiveness, and impact of PA and NP postgraduate program accreditation remain largely unanswered, this study’s findings indicate interest among PA and joint PA/NP postgraduate programs in achieving accreditation for program validation and assessment of educational quality. Furthermore, the results seem to suggest that postgraduate programs believe accreditation will give them a competitive edge in attracting prospective applicants. Therefore, unaccredited postgraduate programs may find themselves at risk for competitive disadvantage in the future. Given the increased emphasis on documenting outcomes and assuring a basic level of quality, postgraduate PA and NP residency/fellowship programs may find significant value in achieving accreditation.

## References

[1] Kidd VD, Vanderlinden S, Hooker RS (2021). A national survey of postgraduate physician assistant fellowship and residency programs. BMC Med Educ.

[2] Kidd VD, Vanderlinden S, Spisak JM (2021). An analysis of the selection criteria for postgraduate physician assistant residency and fellowship programs in the United States. BMC Med Educ.

[3] Kesten KS, El-Banna MM, Blakely J (2019). Educational characteristics and content of postgraduate nurse practitioner residency/fellowship programs. J Am Assoc Nurse Pract.

[4] Park J, Covelli AF, Pittman P (2021). Effects of completing a postgraduate residency or fellowship program on primary care nurse practitioners' transition to practice. J Am Assoc Nurse Pract.

[5] (2023). The consortium receives federal recognition as accrediting agency by the U.S. Department of Education. https://www.apppostgradtraining.com/2022/01/31/the-consortium-receives-federal-recognition-as-accrediting-agency-by-the-u-s-department-of-education/.

[6] Cosme S (2023). Elevating advanced practice provider fellowships through accreditation. J Contin Educ Nurs.

[7] (2023). Announcing ACEN/ARC-PA joint accreditation for Advanced Practice Provider Postgraduate Program and call for comments on standards and policies. https://www.acenursing.org/joint-accreditation-app/.

[8] Maura N. Polansky, J. Barton Gillum, Maxwell S. Rooney PA/NP postgraduate clinical training: a novel model of integrated, interprofessional development. Jonathan Messing, MSN, ACNP-BC, PA/NP Postgraduate Clinical Training: A Novel Model of Integrated, Interprofessional.

[9] Philibert I, Blouin D (2020). Responsiveness to societal needs in postgraduate medical education: the role of accreditation. BMC Med Educ.

[10] Kidd VD, Spisak JM, Vanderlinden S, Kayingo G (2022). A survey of implicit bias training in physician assistant and nurse practitioner postgraduate fellowship/residency programs. BMC Med Educ.

[11] White M, Cosme S, Drown S (2021). Revisiting the impact of accreditation on transition to practice programs: findings from a replication analysis. J Contin Educ Nurs.

[12] Brown K, Poppe A, Kaminetzky C, Wipf J, Woods NF (2015). Recommendations for nurse practitioner residency programs. Nurse Educ.

[13] Hussaini SS, Bushardt RL, Gonsalves WC (2016). Accreditation and implications of clinical postgraduate PA training programs. JAAPA.

[14] Hart AM, Seagriff N, Flinter M (2022). Sustained impact of a postgraduate residency training program on nurse practitioners' careers. J Prim Care Community Health.

[15] Kidd VD (2022). An evaluation of the postgraduate physician assistant/associate and nurse practitioner orthopedic surgery fellowship and residency websites in the United States. Cureus.

[16] Kidd VD, Hooker RS (2021). Postgraduate programs in orthopaedic surgery for physician assistants and nurse practitioners. Orthop Nurs.

[17] Akdemir N, Malik R, Walters T, Hamstra S, Scheele F (2021). Clinicians' perspectives on quality: do they match accreditation standards?. Hum Resour Health.

[18] (2023). Emergency medicine physician assistant postgraduate education program standards. https://www.sempa.org/globalassets/sempa/media/pdf/about-sempa/2021-sempa-postgraduate-program-standards---august-2021.pdf.

[19] Wu F (2019). Emergency medicine physician assistant (EMPA) postgraduate training programs: program characteristics and training curricula. West J Emerg Med.

[20] (2023). Association of postgraduate PA programs (APPAP): criteria of membership. https://appap.org/membership/criteria/.

[21] Mumford V, Greenfield D, Hogden A, Forde K, Westbrook J, Braithwaite J (2015). Counting the costs of accreditation in acute care: an activity-based costing approach. BMJ Open.

[22] Cook C, Heath F, Thompson RL (2000). A meta-analysis of response rates in web- or internet-based surveys. Educ Psychol Meas.

